# Russian Orthodox framing of abortion in online journalism on religion

**DOI:** 10.1177/00377686231198783

**Published:** 2023-10-16

**Authors:** Caroline HILL

**Affiliations:** Uppsala Universitet, Sweden

**Keywords:** abortion, framing, morality policy, online media, Russian Orthodox Church, avortement, encadrement, Église orthodoxe russe, médias en ligne, politique de la morale

## Abstract

When making public statements about abortion, those serving in the Russian Orthodox Church are beholden to the legacy of the Soviet health care system and the need to connect with audiences whose religious sentiments are largely nominal. This article explores framing of abortion by clerics and others serving in the Church in 150 Russian online newspaper articles. Said framing was analyzed according to typologies from prior research of morality policy and church-state relations in Russia. The frequency with which these frames were employed was measured and cross-referenced with article genres. The results show that rational-instrumental frames rooted in secular reasoning surpassed religious argumentation and appeals for state intervention, and that frames expressing disillusionment with the Russian government outpaced positive assessments of the state.

## Résumé

Lorsqu’ils font des déclarations publiques sur l’avortement, les membres de l’Église orthodoxe russe ont à assumer l’héritage du système de santé soviétique et la nécessité de se rapprocher d’un public dont les sentiments religieux sont en grande partie nominaux. Cet article explore le cadrage de l’avortement par des religieux et d’autres personnes servant dans l’Église dans 150 articles de journaux en ligne russes. Ce cadrage a été analysé selon des typologies issues de recherches antérieures sur la politique de la morale et les relations entre l’Église et l’État en Russie. La fréquence d’utilisation de ces cadres a été mesurée et croisée avec les genres d’articles. Les résultats montrent que les cadres rationnels-instrumentaux ancrés dans le raisonnement séculier ont dépassé l’argumentation religieuse et les appels à l’intervention de l’État, et que les cadres exprimant une désillusion à l’égard du gouvernement russe ont dépassé les évaluations positives de l’État.

## Introduction

In the years following the collapse of the Soviet Union, the Russian Orthodox Church^1^ as an institution and the clerics and others serving therein have established themselves as participants in morality policy debates. When doing so, ROC actors strive to adopt framing strategies that will resonate not only with the dedicated believers who comprise a sliver of the Russian population (Yemelyanov, 2018), but with mainstream audiences whose ties to the Church are purely nominal. In the case of debates concerning regulation of access to abortion, Church speakers face an additional challenge: the legacy of the Soviet health care system, where abortion was legalized early and utilized as a preferred birth control method for decades (Luehrmann, 2017).

This article analyzes argumentation by clerics and others^2^ serving in the ROC in articles on the Web sites of popular newspapers, with the goal of determining which morality frames (Hill, 2021; Mucciaroni, 2011) are employed: religious, rational-instrumental, and/or procedural. Procedural frames are evaluated further to determine whether frames describing church-state relations are used, from least to most critical of the state: symphony, affinity, disillusionment, and disestablishment (Hill, 2021; Stoeckl, 2018). These results are then cross-referenced by article genre (building on Lövheim and Lundby (2013) and Lundby et al.’s (2018) studies) in order to establish what frames ROC actors are more likely to use in article types where they have greater editorial agency.

In doing so, this article aims to answer the following question: ‘What frames are employed by clerics and others serving in the Russian Orthodox Church when commenting on abortion in media genres with varying levels of editorial control?’ By increasing understanding of the communication strategies of the Russian Orthodox Church on abortion when addressing a mostly secular audience, the results are expected to contribute to studies of framing and concepts of morality in political science, and mediatization in sociology of religion.

## Abortion in Russia

In the early Soviet years, the Bolsheviks legalized abortion in 1920, ‘making revolutionary Russia the first modern state that made the procedure available to women on demand at medical institutions’ (Luehrmann, 2017: 104). Although the state did not seek to promote abortion as a birth-control method, Soviet women were confronted with chronic shortages of alternative contraceptive methods and deliberate attempts by the Soviet Health Ministry to discourage their use as part of top-down, pro-natalist policies (Johnson, 2004; Luehrmann, 2017; Rivkin-Fish, 2018). For decades, abortion became ‘an accessible and socially accepted, though painful, way to regulate family size and space births’ (Luehrmann, 2017: 105).

Russia’s transition to a market economy in the 1990s was credited with a decrease in abortion rates in the country (Rivkin-Fish, 2018; Sakevich and Denisov, 2019), as ‘private health care providers, Western pharmaceutical companies, commercial mass media, international foundations and agencies, new nongovernmental organizations and the Russian Orthodox Church – began to play a role in family planning’ (Regushevskaya et al., 2009: 51). The first post-Soviet restrictions on abortion were adopted in 2003 and 2007, when the list of ‘social criteria’ for obtaining a late-term abortion was reduced (Rivkin-Fish, 2017: 1734). In 2011, a mandatory waiting period for abortions (which can encapsulate anti-abortion counseling and other interventions) and provisions for doctors who refused to perform terminations (‘conscientious objectors’) were introduced (Ministry of Health of the Russian Federation, 2011).

Despite these measures, Russian abortion laws are relatively permissive: terminations performed within the first 12 weeks of gestation ‘must be provided within the framework of the basic Mandatory Medical Insurance program – meaning, free of charge’ (Sakevich et al., 2016: 463). Although live births have surpassed abortions since 2007 (Denisov and Sakevich, 2014: 52), concern over the number of terminations performed remains: in 2020, Deputy Minister of Health Oleg Salagay stated that around 523,000 abortions were performed the previous year, and pronounced these figures ‘still very high’ (TASS, 2020).

## Opposition to abortion: the Russian Orthodox Church

The fall of the Soviet Union gave the Russian Orthodox Church opportunities to join debates of ‘issues of social ethics and public morality’, including abortion (Stoeckl, 2020: 45). A key step in this process was the publication of *The Basis of the Social Concept of the Russian Orthodox Church* in 2000, hereinafter referred to as *The Basis of the Social Concept* (Russian Orthodox Church, 2008). In Section XII thereof, ‘Problems of Bioethics’, the document stated that abortion was ‘a serious sin (…) the birth of a human being is a gift from God; therefore, from the moment of conception, any attempt on the life of a future human individual is criminal’ (Russian Orthodox Church, 2008). *The Basis of the Social Concept* marked the start of increased efforts by the Church to lobby against abortion before the Russian state authorities and general public.

When doing so, those in the Russian Orthodox Church are forced to confront ‘abortion culture’, a legacy of the ubiquity of abortion within the Soviet health care system (Johnson, 2004; Karpov and Kääriäinen, 2005; Rivkin-Fish, 2017). This is described by Karpov and Kääriäinen (2005: 14) as ‘the widespread and deep-seated view that abortion is a perfectly acceptable way of dealing with medical and socioeconomic hardships in personal and family life’. According to Luehrmann (2017: 106), ‘abortion was rarely regarded as a “right”’ during the Soviet period; instead, women saw it ‘more as an unpleasant duty’ and ‘one of the inevitable hardships of women’s lives’. Those who wish to campaign against abortion in Russia must bear in mind that a large share of their female audience has terminated a pregnancy (or multiple pregnancies) under circumstances in which they may have had little agency.

In addition, those serving in the Church must contend with another facet of the Soviet legacy: what Karpov and Kääriäinen (2005: 25) termed the ‘ruthless destruction of the nation’s religious heritage’, which led to a decrease in religious belief and observance among the population. Although the percentage of Russians who nominally identify as Orthodox doubled from the twilight of the Soviet Union in 1990 to 2020 (from 33% to 68%; Levada-Center, 2020), the share of those who fit Yemelyanov’s (2018: 35, 44) definition of ‘practicing Orthodox Christians (…) those who take Communion once per month [or] more’ has remained around 3%. For those trying to testify against abortion in the Orthodox Church, this means that religious arguments regarding personal culpability and sin could be a hard sell.

## Theoretical approaches to morality framing

The term ‘morality policy’ was initially used to refer to a category of policies that ‘seek to regulate social norms or which evoke strong moral responses from citizens’ (Mooney and Lee, 1995: 600), involve ‘questions of first principle’ (Mooney, 1999: 675), or ‘engage questions of what is right and wrong’ in disputes that ‘are embedded in conceptualizations of ethics and faith’ (Hollander and Patapan, 2016: 1). This term was later expanded to encompass ‘successful agenda setting and framing by interest groups’ in the West, which included ‘economic and public health’ arguments (Studlar, 2008: 394). Mucciaroni (2009: 13�14.) classified frames based on whether they addressed ‘deontological principles’, ‘social consequences’, or ‘procedures’ that the state should implement.

These categories were later refined by Mucciaroni (2011: 211) into ‘morality talk’ based on moral and religious principles, ‘rational-instrumental frames (…) calling attention to the negative consequences for society’, and ‘procedural terms’ that concerned ‘how policymakers should make decisions, which level of government or institution should properly make them, and whose preferences should be weighed the most heavily’ when doing so. Conservative actors may lean toward rational-instrumental and procedural framing because it ‘involve[s] less political risk’ when compared to arguments ‘that pass moral judgement’ (Mucciaroni, 2011: 210).

Existing research of anti-abortion activism in Russia has revealed a tendency toward rational-instrumental argumentation. Rivkin-Fish (2013, 2017) found that nationalist and other conservative groups placed emphasis on demographics and framed contraception and family planning programs introduced after the fall of the Soviet Union as ‘a cynical Western ruse to further Russia’s population decline by convincing women to refuse childbearing’ (Rivkin-Fish, 2013: 573). In a similar vein, Mason (2019: 679) noted the limited utility of religious framing in a country that had experienced decades of state atheism and found a preference for frames that highlighted demographic concerns ‘about losing the nation to those who may outbreed the Russians’.

A prior analysis of clerics and others serving in the ROC via Russian Orthodox online news portals (Hill, 2021) employed a morality frame typology based on Mucciaroni (2011) and adapted to the Russian case. The results showed that rational-instrumental frames dominated; however, unlike Mucciaroni’s (2011) findings, procedural frames lagged behind religious arguments by a significant margin. In addition, four procedural sub-frames based on Stoeckl’s (2018) models of church-state relations were identified: ‘symphony’ frames promoting church-state partnerships, ‘affinity’ frames expressing approval of state initiatives, ‘disillusionment’ frames expressing frustration with the state’s performance, and ‘disestablishment’ frames portraying the state as opposed to or threatening the general population’s well-being (Hill, 2021: 6, 7).

In the Russian Orthodox online portals, disillusionment frames were used most frequently, followed by affinity frames; symphony frames were a distant third, and disestablishment frames expressing open hostility to the state were rarely employed (Hill, 2021). Nonetheless, the overall dearth of procedural frames compared to shares found in previous morality policy studies in the West indicates that the case of ROC speakers and attitudes thereof toward the Russian state merits further exploration.

## Framing, journalism on religion, and media genre

When engaging in morality framing before a national audience, conservative public figures must consider not only the greater audience that they hope to reach but also the forms and genres of media through which this is accomplished. In his study of Christian groups in the United Kingdom, Kettell (2017: 287) noted the importance of audience when making morality policy arguments, as framing ‘must appeal to two distinct “internal” and “external” sets of audiences (generally breaking down into group and non-group members)’.

Within mediatization research of the Nordic countries, the aforementioned ‘internal’ audience is catered to by what Hjarvard refers to as ‘religious media’, whereas ‘external’ audiences are more likely to experience religion through what Hjarvard (2012: 28) labels ‘journalism on religion’, which ‘subjects religion to the dominant discourses of the political public sphere’. According to Hjarvard (2012: 33), media within the form of journalism on religion restrict ‘the ability of organized religious organizations and individuals to define and frame religious issues in the public sphere and they are subsequently much more exposed to public criticism based on the general social and political norms of secular society’.

Prior research of mediatization in the context of the Russian Orthodox Church has focused largely on the Church and its members’ own religious media ventures (Grishaeva, 2020; Grishaeva and Shumkova, 2018; Ostrovskaya, 2021; Ponomariov, 2015; Staehle, 2018). This article shifts the empirical focus to online secular journalism on religion and draws upon Lövheim and Lundby (2013) and Lundby et al.’s (2018) studies of religion in various article genres in the Nordic countries: Lövheim and Lundby (2013: 31, 30) found that genres such as ‘[n]ews and features on religion are subject to extensive editorial formatting and may thus be given a more mediatized form’ than the genres of ‘religious columns’ and ‘editorials’, while Lundby et al. (2018: 206) determined that even in ‘religious columns’ authored by clergy members, ‘content is rarely explicitly religious’. The current study is an exploration of how ROC actors conform to editorial norms of Russian journalism on religion and genres therein.

## Materials and methods

The sample for this article was assembled through a keyword search^3^ covering a period starting in 2000, the year that *The Basis of the Social Concept* was published, and ending in 2020, the year that changes to the Russian Constitution were made and ‘roles for the Church inside society as well as vis-à-vis the Russian state’ had stabilized in an ‘end-point to the previous, volatile period’ following the fall of the Soviet Union (Stoeckl, 2020: 3, 9). The queries were made on the Web pages of 11 Russian newspapers: *Argumenty i Fakty* (aif.ru), *Izvestiya* (iz.ru), *Kommersant* (kommersant.ru), *Komsomolskaya Pravda* (kp.ru), *Mir Novostey* (mirnov.ru), *Moskovskiy Komsomolets* (mk.ru), *Nezavisimaya Gazeta* (ng.ru), *Novaya Gazeta* (novayagazeta.ru), *Parlamentskaya Gazeta* (pnp.ru), *Rossiyskaya Gazeta* (rg.ru), and *Vedomosti* (vedomosti.ru). These sources were selected according to their placement in rankings compiled by the Medialogiya Informational-Analytical System.^4^

**Figure 1.**
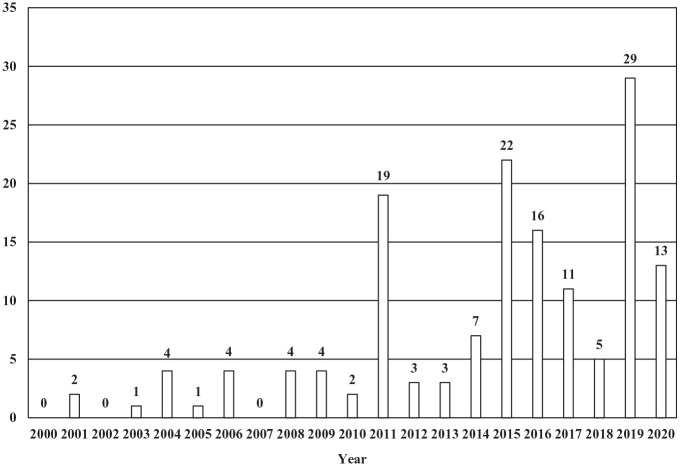
Articles by year (total *n* = 150).

Those articles that included the keywords and were authored by or contained quotes from clerics and others serving in the Church regarding abortion were compiled in a data set of 150 articles (see Figure 1). Of the articles accumulated, 8 were clerical opinion pieces, 3 were clerical Q&A pieces, 21 were interviews with clerics, 34 were features on religion, and 84 were news pieces on religion. Years where higher numbers of articles were found included 2011, when new restrictions were placed on abortion access (Ministry of Health of the Russian Federation, 2011); 2015, when Patriarch Kirill addressed the Russian Duma as part of the Parliamentary Christmas Meetings (*Argumenty i Fakty*, 2015); and 2019, the year that the ROC released a draft document titled ‘On the Inviolability of Human Life from the Moment of Conception’ (Russian Orthodox Church, 2019).

The individuals who authored articles or were quoted were predominantly clerics (a total of 48). Two individuals serving in the Russian Orthodox Church who were not clerics were included in the sample: Abbess Kseniya (Chernega),^5^ head of the Legal Service of the Russian Orthodox Church, and Vitaliy Milonov, a deputy in the Russian State Duma and altar server and subdeacon in the years surveyed. These individuals were included in the analysis after a pilot study of the materials found that their framing strategies mirrored those of ordained clerics.

### Deductive analysis

The articles located were subjected to a deductive analysis in which it was first determined to which genre they belonged. Genres were ranked in order of degree of editorial influence, or the extent to which ROC actors might be compelled to conform to secular media logic (from least to greatest): clerical opinion pieces, clerical Q&A (similar to the ‘ask the priest’ genre within online religious media studied by Bogdanova, 2020), interviews with clerics, features on religion, and news on religion. The texts thereof were coded by overarching morality frame categories (religious, rational-instrumental, and procedural), and articles where procedural frames were found were also coded for church-state relations frames (symphony, affinity, disillusionment, and disestablishment). The total number of times that each frame type occurred was counted, and the morality and church-state relations frames used the most often were labeled as dominant for each article; in the event that two frame types were within 5% of each other, the article was double-coded. Dominant frames and article genres were then cross-analyzed in order to determine which frames were used most frequently by genre.

## Results

In this section, results will be reported for morality frames and church-state relations frames within the procedural frame category, respectively; first in terms of dominant frames reported across all genres, then according to genre. The most common arguments within the framing categories will then be presented.

### Morality framing

**Figure 2.**
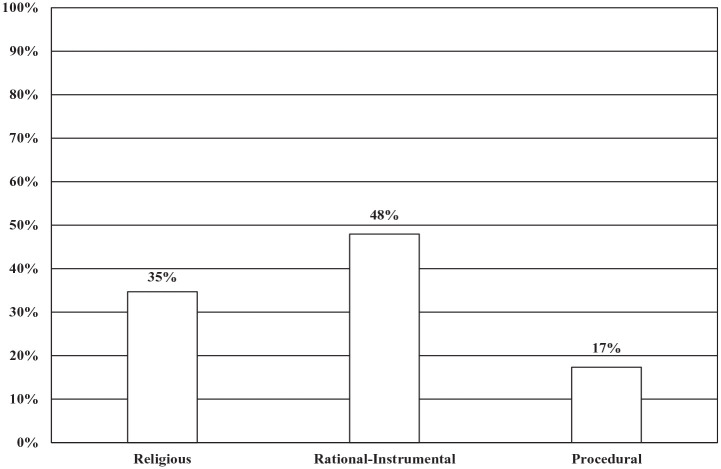
Dominant morality policy frames (*n* = 150).

When the dominant frames across all genres were compiled, 48% (94 articles) of the articles analyzed had dominant rational-instrumental frames, followed by religious dominant frames at 35% (68 articles) and procedural frames at 17% (34 articles) (see Figure 2).

### Morality framing by genre

**Figure 3.**
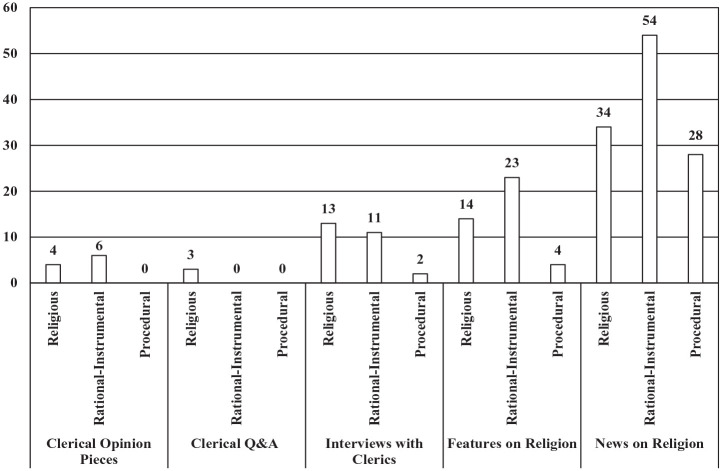
Dominant morality policy frames by article genre (*n* = 150).

Among the clerical opinion pieces, rational-instrumental frames led with six articles, followed by religious frames with four articles; none of the articles had a dominant procedural frame. This contrasted with clerical Q&A pieces, where the three articles found had a dominant religious frame, and interviews with clerics, where religious frames held first place with 13 articles, rational-instrumental frames came in second with 11 articles, and procedural frames held third place with 2 articles. Features on religion were mostly rational-instrumental with 23 articles, with religious dominant frames in second place with 14 articles, and procedural frames in third place with 4 articles. Among news pieces on religion, rational-instrumental dominant frames were in first place with 54 articles and religious dominant frames were in second with 34 articles, but procedural dominant frames held a much larger share than among other article genres with 28 articles (see Figure 3).

### Religious frames

In the sections to follow, arguments found within the framing categories will be presented. Religious framing mostly focused on the Russian Orthodox Church as an institution, abortion as a sin, God and the role thereof in Russians’ lives, abortion as murder, and other religious groups’ beliefs regarding abortion. Frames concerning the Orthodox Church focused on the ROC’s beliefs on abortion and role in Russian society. In the case of the former, ROC declarations regarding the humanity of the embryo and fetus were key: Bishop Panteleimon (Shatov) of Orekhovo-Zuyevo contended, ‘The Church believes that an immortal soul appears in every child already in the mother’s womb. Therefore, we cannot accept that living people are perceived as garbage’^6^ (Bishop Panteleimon, 2016). Others framed the Church as a conservative entity in morality policy debates: Metropolitan Sergiy (Fomin) of Solnechnogorsk argued,

The Church is called upon to be conservative (…) the Orthodox Church will not start [to perform] same-sex marriages, will not introduce the institution of female priesthood, will not be indulgent toward prostitution and propaganda thereof, will not agree with abortions. (Yakovleva, 2003)

When abortion was labeled as a sin, it was portrayed as unique in terms of gravity: anti-abortion activist Priest Vladimir Dukhovich opined, ‘This sin is irreversible. If you stole something, you can return it; if you deceived someone, you can apologize and tell the truth. It doesn’t work like that with murder’ (Ovchinnikov, 2018). Speakers also touched on contrasts between the ROC’s stance and the general population’s attitudes toward abortion: Bishop Isidor (Tupikin) of Smolensk and Vyazemsk proclaimed that ‘the Church constantly says that abortion is a sin, but society only listens to what it wants (…) and does not react to the calls that the Church sends out about such serious issues’ (Yefremova, 2014).

References to God typically centered on the unborn as a gift thereof. Archpriest Vladimir Vedernikov used this argument to advocate for bringing pregnancies to term, even in the event of fetal pathologies: ‘God cannot give us anything out of spite. Including a sick child. It is all for our benefit and for our salvation’ (*Argumenty i Fakty*, 2006). The concept of the fetus as God’s gift was also utilized as a warning to those who might terminate pregnancies: Priest Vladimir Dukhovich declared that ‘[abortion] is, in fact, spit[ting] in the face of God. Because only God gives life on earth. If we reject [life], we reject the Creator’ (Chinkova, 2011).

Framing of abortion as murder overlapped heavily with characterizations of abortion as sinful and the degree to which it might be pardonable by the ROC. While Priest Vladimir Dukhovich contended that ‘the truth of this matter is that this is just murder (…) no exceptions exist. Not the period [of gestation], nothing can justify this situation at all’ (Chinkova, 2011), Priest Arkadiy Makovetskiy granted that ‘abortion for medical reasons is also the murder of an infant in the womb, although [it is] more excusable’ (Kalyuzhnaya, 2017).

References to other religious groups focused primarily on common beliefs regarding abortion: Patriarch of Moscow and All Rus Kirill affirmed, ‘Practically every traditional religion (…) does not allow abortions. And not only monotheistic, but pagan’ (Yakovleva, 2016). In a surprising turn, these arguments also included unflattering comparisons of Russian Orthodox Christians with followers of other religions, such as Archpriest Andrey Tkachyov’s commentary on Islam and birth rates:

Regardless of what kind of faith Muslims have, they have it. It nourishes them, it enlivens them. And therefore, youth obey elders, a woman obeys [her] husband. A woman gives birth to many children and does not have abortions. Any Islamic country is better than us, because we just murder millions of people in the womb every year. (Baranov, 2016)

### Rational-instrumental frames

When employing rational-instrumental frames, the actors studied made arguments that primarily concerned science, medicine, demographics, economic issues, law, and abortion as murder. Science was used to contend that a fetus was a separate entity from a woman’s body: Subdeacon and parliamentary deputy Vitaliy Milonov charged that

In studies by the [Moscow State University] embryology department, it is clearly stated: a child cannot be considered part of the mother’s body, because a body part cannot be a different sex, cannot [have] a different blood type (…) and we are simply obligated to agree with scientific opinion. (Ivanova, 2015)

Abbess Kseniya (Chernega), head of the legal department of the Moscow Patriarchate, combined rational-instrumental and procedural frames when calling for the country’s regulation of abortion to be tightened – and referenced international practices:

In the laws of 119 countries, it is stated that the basis for termination of pregnancy can only be those medical indicators that threaten the health and life of a pregnant woman (…) it must be recognized that an unborn child is not a part of the mother’s body, but a person [who] has, like all people, the right to life. (*Kommersant*, 2019b)

In the event that clerics had a previous career in medicine or science, this was used as a supporting argument for rational-instrumental claims. When arguing that human life began at conception, Priest Vladimir Dukhovich stated that ‘I [have a PhD] in biological sciences (…) and many of my colleagues, scholars of embryology, also support this point of view’ (Ovchinnikov, 2018). Archpriest Pyotr Guryanov similarly noted that ‘I have been collaborating with women’s clinics for 10 years already; I have a permit [to do so] and medical education as a military surgeon’ (*Kommersant*, 2019a).

References to the country’s demographic situation highlighted the number of abortions performed per year. Bishop Panteleimon (Shatov) cited official statistics from the Russian Ministry of Health: ‘627 thousand abortions were recorded in 2017. And although this is less than in previous years (in 2010, more than 1 million abortions), this is a horrifying number nonetheless’ (Bishop Panteleimon, 2018). Abortions performed outside of state-run medical facilities were presented as evidence that the number of procedures was higher, with Bishop Panteleimon (Shatov) charging,

Official statistics are based on data of state and municipal clinics. It is understood that this is just the tip of the iceberg. It’s difficult to even imagine how many murders of infants take place in private facilities, and how many medication abortions are performed with no monitoring whatsoever. (Bishop Panteleimon, 2016)

Economic arguments were centered on use of state funds and fused with procedural claims on behalf of taxpayers. Patriarch Kirill declared that ‘I consider the removal of operations for artificial termination of pregnancy from the system of mandatory medical insurance, which is supported at the expense of taxpayers, including those who categorically do not accept abortions, to be morally justified’ (*Argumenty i Fakty*, 2015). Head of the Saratov Archdiocesan Society of Orthodox Doctors Priest Sergiy Klyayev similarly argued that ‘if a woman has decided to have an abortion, she must pay for it herself’ (Likhoman, 2011).

Legal frames were similarly entwined with procedural arguments, particularly concerning the human rights of children. Abbess and lawyer Kseniya (Chernega) reasoned, ‘In the Convention on the Rights of the Child, it is said that a child is in need of special protection and care (…) including proper legal protection both before and after birth’, and proposed including similar text into Russian federal law on child rights (*Nezavisimaya Gazeta*, 2019). In addition, Abbess Kseniya noted that ‘Russian inheritance legislation recognizes the human fetus as a legal subject’ when lobbying for the strengthening of legal conditions that would aid future efforts to ban abortion (*Kommersant*, 2019b).

Rational-instrumental claims that abortion equaled murder often referenced the human toll exacted by World War II (WWII) and Nazism on Soviet Russia. Archpriest Dimitriy Smirnov referred to ‘Eternal Regiment’ parades (in which the descendants of fallen soldiers from WWII march with photos of their ancestors) when arguing, ‘We attend “Eternal Regiment” [marches]. But enemies killed them. How many have we killed ourselves[?] (…) the mass murder of Russian children by Russian people is more frightening than the Holocaust’ (Khovanskaya, 2019b). Patriarch Kirill chose a similar tack when arguing against abortions due to fetal pathologies, remarking that

[By] killing a fetus that is imperfect in some way, we kill an invalid. Only Hitler did such a thing (…) tweaking the human race just because an embryo won’t turn into a good lawyer is a crime against humanity. (Khovanskaya, 2019a)

### Procedural and Church-State relations frames

**Figure 4.**
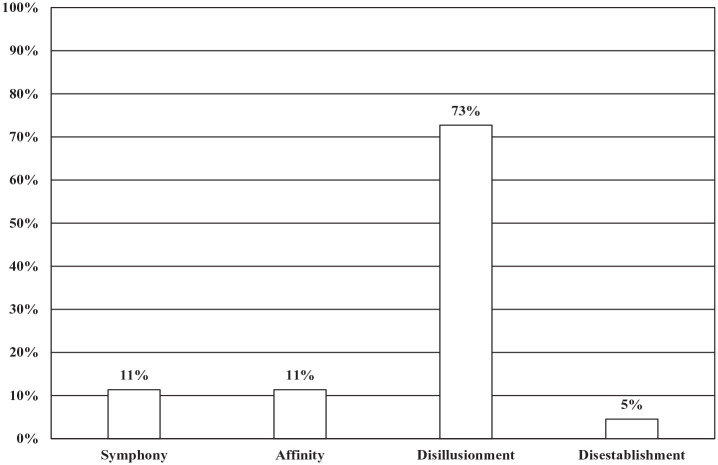
Dominant church-state relations frames (*n* = 42).

Statements that were found to use procedural framing were analyzed further for church-state relations frames; in total, 42 articles featured church-state relations framing. Of these, 73% (32 articles) had dominant disillusionment frames in which speakers expressed disappointment with state policy or the behavior of civil servants, and symphony and affinity frames lagged behind with a distant 11% (5 articles) each; disestablishment frames were in last place with only 5% (2 articles) (see Figure 4).

**Figure 5.**
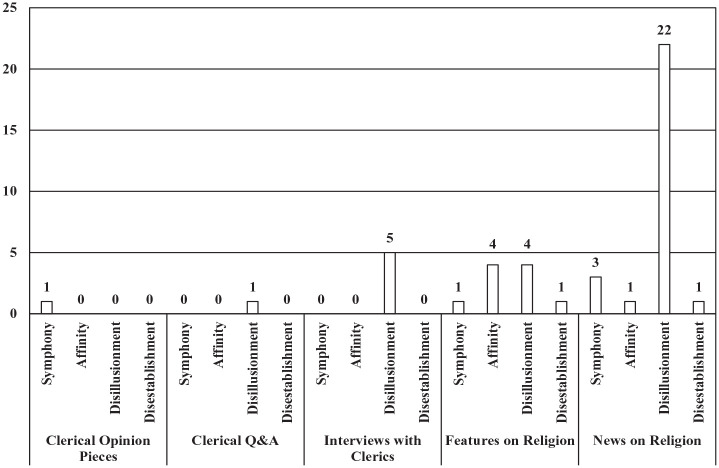
Dominant church-state relations frames by article genre (*n* = 42).

When the results were compared by article genre, it was found that the one clerical opinion piece that included church-state framing had a dominant symphony frame. Among clerical Q&A pieces, the one article found to have church-state framing had a dominant disillusionment frame; the same pattern was found among interviews with clerics, with all of 5 articles showing dominant disillusionment frames. Features on religion showed varied results, with affinity and disillusionment dominant frames tied with 4 articles each, and symphony and disestablishment frames tied with one article each. Among news articles on religion, disillusionment dominant frames led with 22 articles, while symphony frames were in second place by a large margin with 3 articles, and affinity and disestablishment frames tied with only one article each (see Figure 5).

### Symphony frames

Framing that highlighted successful partnerships between church and state focused mostly on crisis pregnancy centers and other abortion prevention initiatives. In the early 2000s, Archpriest Dimitriy Smirnov spoke of cooperation between medical staff and his parish:

We made an agreement with a maternity hospital and opened a contact point there, and the head physician permitted us to speak with women who had come to kill their children. We managed to talk every tenth out of [committing] murder. (Konstantinov, 2001)

Years later, Archpriest Pyotr Guryanov reported a similar initiative:

A girl comes to get a referral for an abortion, [and] the gynecologist suggests that she has a chat with a mullah or an Orthodox Christian priest, depending on religious confession (…) as a result of our efforts, we have saved almost 300 children. (*Kommersant*, 2019a)

References to fruitful teamwork between the ROC and the state amounted to the most optimistic framing of the Russian government’s actions.

### Affinity frames

Those clerics who spoke positively of the work of state authorities and employees focused primarily on gradual improvements in state policy. At an awards presentation for women’s clinics as part of an anti-abortion campaign, Bishop Isidor (Tupikin) remarked that ‘It is pleasant to see that there is an understanding at all levels of state power of how it is necessary to unite in the fight against this misconduct’ (Yefremova, 2014). For his part, Patriarch Kirill noted changes in legislation when visiting a shelter for at-risk mothers on Easter: ‘In our society, including at the level of the state (…) positive shifts are taking place. And today, at least, we don’t encounter open propaganda of abortion’ (Khovanskaya, 2020). Despite these signs of optimism, affinity frames were overshadowed by critical portrayals of the state.

### Disillusionment frames

Clerics and others serving in the Church mostly expressed disappointment with the work of state authorities: inadequate welfare benefits, and the attitudes of politicians toward the ROC’s involvement in debates on abortion. Patriarch Kirill was skeptical of state measures aimed at ‘support of motherhood’, opining that it was ‘wonderful that we have maternity benefits, wonderful that the state takes some kind of steps, but these are miserly steps’ (*Izvestiya*, 2019). Then-regional Deputy Vitaliy Milonov alleged harassment from other deputies as retribution for promoting anti-abortion initiatives, using religious terms: ‘I was simply crucified. I was showered with curses. And I [sat] (…) so thankful that the Lord deemed me worthy of suffering for a good initiative’ (Ivanova, 2015). While the speakers criticized the state’s failures to provide social welfare and aired grievances against legislators, this framing fell short of total enmity between the Church and state.

### Disestablishment frames

In the few cases where hostile framing of the state was found, ROC clerics primarily focused anger on the role of the state as a provider of abortion services, and funding thereof through the state budget. Archpriest Dimitriy Smirnov placed blame on the state for financing abortions while expressing anxiety over the country’s demographics, opining that ‘it is particularly disgusting that the state itself participates in all of this (…) we have turned into a people [that are] dying out, and the state has decided to pay for the murder of its own citizens’ (Tsikulina, 2016). While the wording of such critiques was strident, they were outpaced by disillusionment frames with a softer tone.

## Discussion and conclusion

This article has analyzed framing employed by Russian Orthodox clerics and others serving in the Church when communicating their opinions on abortion via Russia’s most popular online newspapers. On one hand, the results mirrored those found in prior research of morality policy issues (Mucciaroni, 2011) in terms of the dominance of rational-instrumental framing over religious arguments. In addition, the findings for articles wherein ROC actors had the most editorial control, clerical opinion pieces, echoed those of Lundby et al.’s (2018) results for religious columns: even when religious actors are granted more freedom to structure their arguments as they wish, they may eschew religious framing.

The results diverged from prior studies of morality policy in the West in that procedural frames lagged far behind both rational-instrumental and religious arguments, showing a reluctance on the part of the clerics and others quoted here to make demands of or publicly oppose the state. In this way, the results were consistent with the previous analysis of framing via Russian Orthodox news portals (Hill, 2021).

While all of the actors quoted in this analysis were opposed to abortion in principle, future analyses in the form of qualitative interviews could delve more deeply into the extent to which clerics would consider abortion pardonable under certain conditions (risk to the mother’s life, for example). This would provide insight into the difference between public communication strategies and pastoral care, as well as the diversity among clerics in terms of conformity with *The Basis of the Social Concept* (Russian Orthodox Church, 2008).

The results for framing of church-state relations differed significantly from the prior study of framing of abortion in Russian Orthodox news portals (Hill, 2021), as disillusionment frames led by a large margin over all others. Given the history of state repression against the Russian Orthodox Church and continued state funding of abortions, one might assume that clerics would feel more comfortable making negative assessments of the work of state organs via religious media, a venue that Kettell (2017) would term an ‘internal’ audience. However, the results found here were just the opposite: there was much more acerbic commentary via mainstream media sources geared toward an ‘external’ public.

When cross-analyzed by article genre, there was considerable variation in terms of framing under varying levels of editorial control – a finding consistent with Lövheim and Lundby’s (2013) comparison of article genres. It is remarkable that clerics and others serving in the ROC opted for mostly rational-instrumental framing when authoring opinion pieces, but clerical Q&A pieces and, to a lesser degree, interviews with clerics featured more religious dominant frames. This may be an effect of clerics and others assuming the role of spiritual advisor within these genres, or what Bogdanova (2020: 207) termed ‘mediatization of pastoral care’.

The absence of dominant procedural frames for any genre other than those with greater editorial control (interviews with clerics, feature articles, and news on religion) shows a certain reluctance by individual actors in the ROC to challenge the state authorities on their own. Likewise, the fact that the only genre where clerics used exclusively dominant symphony frames was clerical opinion pieces indicates a greater willingness to criticize the state when the burden of responsibility for messages conveyed is shared by journalists and others in the media production process. This conclusion is further supported by the fact that dominant disestablishment frames that openly accused state authorities of murder or challenged the legitimacy of the state only appeared in the two article genres with the greatest level of editorial control (features on religion and news on religion).

When observing the differences in communication styles between article genres, it is unclear if this is the result of self-censorship by clerics and others in the Church, or editorial agency by journalists and others involved in the publishing process. While prior research (Staehle, 2018) has shown a high degree of awareness on the part of ROC actors of the importance of strategic communication when venturing outside the confines of religious media, the varied results here indicate that further exploration is merited. Additional investigation of micro-level interactions between ROC actors and those in media production (reporters, editors, etc.) with whom they interact on a regular basis, in the form of participant observation or qualitative interviews, would shed light on the balance of power within these exchanges – and the process of morality framing via mainstream journalism on religion.

Another pathway for future research would be to measure the impact of more recent events such as the February 2022 Russian invasion of Ukraine and ongoing war on morality framing of abortion in the ROC. While an incident in which a priest claimed that giving birth to multiple children would mitigate the pain of losing a son in battle attracted media attention (Zakaryan, 2022), a longitudinal analysis of framing in religious media and/or journalism on religion could shift the focus away from sensational utterances and offer greater insight into clerics’ willingness to securitize discussions of abortion.

## Notes

1. Hereinafter also referred to as the ‘ROC’ and ‘Church’.

2. More information on ROC actors studied can be found in the ‘Materials and Methods’ section of this article.

3. Queries for the word combinations ‘аборт* РПЦ’ and ‘аборт* церк*’ were conducted (‘abortion ROC’ and ‘abortion Church’, respectively).

4. https://www.mlg.ru/ratings/media/socmedia/. The Medialogiya Informational-Analytical System ranks Web pages of newspapers and other mass-media sources by the number of hyperlinks to these pages posted on social media per month.

5. The surnames of members of monastic orders are in parentheses, in accordance with Orthodox naming conventions.

6. All translations from Russian to English are the author’s own.
